# Development of a thermostable microneedle patch for polio vaccination

**DOI:** 10.1007/s13346-018-00608-9

**Published:** 2018-12-12

**Authors:** Chandana Kolluru, Yasmine Gomaa, Mark R. Prausnitz

**Affiliations:** 1 School of Materials Science and Engineering, Georgia Institute of Technology, 771 Ferst Drive, Atlanta, GA 30332 USA; 2 School of Chemical and Biomolecular Engineering, Georgia Institute of Technology, 311 Ferst Drive, Atlanta, GA 30332 USA; 3 Department of Pharmaceutics, Faculty of Pharmacy, Alexandria University, El-Khartoum Square, Alexandria, 21521 Egypt

**Keywords:** Dissolving microneedle patch, Inactivated polio vaccine, Thermostability, Thermogravimetric analysis, Differential scanning calorimetry, X-ray diffraction

## Abstract

The aim of this study was to develop a dissolving microneedle (MN) patch for administration of inactivated polio vaccine (IPV) with improved thermal stability when compared with conventional liquid IPV. Excipient screening showed that a combination of maltodextrin and D-sorbitol in histidine buffer best preserved IPV activity during MN patch fabrication and storage. As determined by D-antigen ELISA, all three IPV serotypes maintained > 70% activity after 2 months and > 50% activity after 1-year storage at 5 °C or 25 °C with desiccant. Storage at 40 °C yielded > 40% activity after 2 months and > 20% activity after 1 year. In contrast, commercial liquid IPV types 1 and 2 lost essentially all activity within 1 month at 40 °C and IPV type 3 had < 40% activity. Residual moisture content in MN patches measured by thermogravimetric analysis was 1.2–6.5%, depending on storage conditions. Glass transition temperature measured by differential scanning calorimetry, structural changes measured by X-ray diffraction, and molecular interactions measured by Fourier transform infrared spectroscopy showed changes in MN matrix properties, but they did not correlate with IPV activity changes during storage. We conclude that appropriately formulated MN patches can exhibit thermostability that could enable distribution of IPV with less reliance on cold chain storage.

**Electronic supplementary material:**

The online version of this article (10.1007/s13346-018-00608-9) contains supplementary material, which is available to authorized users.

## Introduction

Poliomyelitis is a highly infectious disease which mainly affects children. The disease can be caused by any of the three serotypes of inactivated polio vaccine (IPV) (type 1, type 2, or type 3) and can be prevented by immunization [1]. Due in large part to the Global Polio Eradication Initiative (GPEI), the number of polio cases has dropped from 350,000 in 1988 to only 22 reported cases in 2017. IPV type 1 is the only serotype that is currently endemic to three countries (Afghanistan, Nigeria, and Pakistan), and wild-type IPV type 2 has been eradicated [2]. However, WHO predicts that failure to eradicate this disease can reverse these advances and cause as many as 200,000 new cases every year in 10 years [3].

The progress made so far is predominantly due to mass vaccination using the oral polio vaccine (OPV) which is a live-attenuated virus given by mouth [4]. OPV offers advantages such as low cost, ease of oral administration, no sharps, and generation of mucosal immunity. However, a major concern with OPV is the possibility of genetic reversion to a virulent form that can cause vaccine-associated paralytic polio (VAPP) [5] and can be transmitted to others. A safer alternative is IPV, which does not carry the risk of paralysis or transmission, but is administered as an intradermal (ID) [6] or intramuscular (IM) injection [7].

To eliminate the risks of VAPP, OPV was replaced by IPV in USA and UK in 2000 and 2004, respectively [8], and plans to eventually discontinue use of OPV worldwide are underway [9]. However, the biggest drawbacks of switching to IPV are the need for trained medical professionals to administer IM or ID injections, generation of sharps waste, need for cold chain storage, high cost of the vaccine, poor mucosal immune responses, and need for multiple doses for a protective immune response [10–12].

In this study, we propose the use of a dissolving microneedle (MN) patch for IPV vaccination. When a MN patch is applied to the skin, micron-scale, solid needles made of safe, water-soluble excipients that encapsulate vaccine penetrate the outer barrier layer of the skin, where they dissolve and release their vaccine payload [13–15]. MN patches can be self-administered painlessly [16–18], generating no sharps waste, and have been used previously for various vaccinations in research [14, 19], including IPV [20], and a recent clinical trials on influenza [21]. Encapsulation of vaccines in appropriately formulated MN patches has been shown to increase vaccine thermostability [22–24]. It was therefore our objective to formulate a MN patch for IPV vaccination that is thermostable, has reduced reliance on cold chain, is easy to administer and eliminates sharps waste. This approach could facilitate mass vaccination campaigns to achieve polio eradication, especially in developing countries [25].

## Materials and methods

### Concentration of inactivated polio vaccine

Monovalent, bulk IPV (Mahoney strain of type 1, Middle East Forces (MEF) of type 2 and Saukett of type 3) was kindly provided by Bilthoven Biologicals (Bilthoven, Netherlands). The initial antigen concentrations were 1675, 963, and 950 D-antigen units (DU)/ml for IPV types 1, 2, and 3, respectively. The bulk IPV was concentrated using Amicon Ultra centrifuge spin filters with 100 kDa MW cutoff (EMD Millipore, Billerica, MA) either ~10-fold by volume to a final antigen concentration of 1.7 × 10^4^, 9.6 × 10^3^, and 9.5 × 10^3^ DU/ml for IPV types 1, 2, and 3, respectively, or ~47-fold by volume to a final antigen concentration of 7.9 × 10^4^, 4.8 × 10^4^, and 4.5 × 10^4^ DU/ml for IPV types 1, 2, and 3, respectively. All D-antigen values were determined by ELISA, as described below.

### Vaccine stability screening

To increase throughput of formulation screening, candidate vaccine-excipient formulations were dried on polydimethylsiloxane (PDMS) chips, which are representative of the surface of a MN patch mold. PDMS chips were prepared by curing a thin film of Sylgard 184 (Dow Corning, Midland, MI) and preparing 6-mm diameter circular discs using a hammer punch. IPV was concentrated 10-fold by volume and mixed with selected excipients. A 10 μl drop of formulated vaccine solution was placed on each chip and allowed to dry at 5 °C in a desiccator filled with desiccant (Drierite, W A Hammond Drierite, Xenia, OH) overnight. After reconstituting the dried IPV in 999 μl media M199, the D-antigen content of the IPV was determined by ELISA, as described below. All excipients were purchased from Sigma-Aldrich (St. Louis, MO).

### Microneedle patch fabrication

PDMS molds consisting of a 10 × 10 array of cavities that form the MNs were fabricated as described previously [26]. The antigen casting solution was prepared by mixing 47-fold concentrated vaccine with a solution consisting of a 10% *w*/*v* maltodextrin and D-sorbitol in 0.35 mM histidine buffer at pH 6.3. Fifty microliters of the antigen solution was cast onto a MN mold, which was then centrifuged for 1 h at 5 °C to fill the mold cavities with solution and partially dried in the mold, following a procedure previously described [27]. This resulted in MN patch loading of 71 ± 1.8 DU, 14 ± 0.6 DU, and 47 ± 3.2 DU of IPV types 1, 2, and 3, respectively, per patch.

The polymer matrix solution used to form the MN patch backing consisted of 35% *w*/*v* fish gelatin and 10% *w*/*v* D-sorbitol in 0.15 mM histidine buffer. The solution was mixed for 1 h at 35 °C until completely dissolved. Two hundred microliters of polymer matrix solution was cast onto a mold pre-filled with partially dried antigen solution. The mold was then placed under vacuum for 2 h at room temperature (20–25 °C) followed by storing in a desiccator at room temperature for 2 days before demolding. Demolded patches were packaged with desiccant in aluminum pouches to maintain 0% RH (relative humidity) and then stored at a given temperature (i.e., 5, 25, or 40 °C) in stability chambers. For experiments involving storage at ~ 50% RH, patches were stored in a desiccator containing a saturated solution of magnesium nitrate in distilled water [28].

### Microneedle composition

The composition of MNs (i.e., just the MNs without the patch backing) was determined by preparing placebo patches (i.e., with no IPV, but otherwise fabricated in the same way) and carefully cutting off the MNs. The MNs were reconstituted in water, and the protein content was determined using micro BCA protein assay kit (Thermo Fisher Scientific, Waltham, MA), which was interpreted to be roughly equal to the gelatin content, since the only protein in a placebo patch should be gelatin from 2nd cast solution which could migrate into the MN during the fabrication process. In this way, the composition of the MNs was estimated to be fish gelatin, D-sorbitol, and maltodextrin in a mass ratio of 55:38:7. This formulation was used when studying IPV stability on chips that simulate MN composition. The chips were stored at a given temperature (i.e., 5, 25, or 40 °C) for specified times in stability chambers.

### Lyophilization

To study the effect of lyophilization on IPV stability, demolded MN patches and dried IPV-excipient chips were subjected to lyophilization (VirTis AdVantage 2.0 BenchTop lyophilizer, SP Industries, Warminster, PA). The lyophilization process involved a primary drying at − 45 °C (10 mTorr) for 3 h followed by a secondary drying at 25 °C (10 mTorr) for 24 h. The samples were removed from the lyophilizer and stored in a desiccator until tested.

### Microneedle patch characterization

MN patches were imaged using brightfield microscopy (Hirox KH-8700, Tokyo, Japan). To assess MN insertion into skin, patches were pressed by thumb with a force of ~ 20 N onto stretched porcine cadaver skin. The MNs were left in the skin at room temperature for 15 min (without further manipulation of the MN patch), and then removed. The skin was stained for 5 min, with gentian violet (2% solution, Humco, Texarkana, TX), which was then wiped off to show preferentially stained sites of MN penetration into skin [29]. The removed MN patches were imaged to determine extent of MN dissolution.

### ELISA assay measurements

The D-antigen content of IPV from chips and MN patches was determined by antigen-capture sandwich ELISA, as previously described [30]. Polio-specific monoclonal antibodies were used for both capture and detection. The capture antibody solutions were prepared by adding type 1 (HYB295-15-02), type 2 (HYB294-06-02), or type 3 (HYB300-05-02) (Thermo Fisher Scientific) specific antibodies to 0.05 M carbonate-bicarbonate buffer, pH 9.6. Type 1 and type 3 antibodies were diluted at 1:1000, and type 2 was diluted at 1:500.

### Thermogravimetric analysis

Thermogravimetric analysis (TGA, Q50, TA Instruments, New Castle, DE) was used to determine the residual moisture content of the MN patches. Samples obtained from MN patch (each weighing 15–20 mg) were heated at 10 °C/min from room temperature to 110 °C and then maintained isothermally for 10 min. The residual moisture content (%) was determined by the stable weight loss (%) at 110 °C using TA Instruments Universal Analysis 2000 software.

### Differential scanning calorimetry

Differential scanning calorimetry (DSC Q200, TA Instruments) was used to measure the glass transition temperature (T_g_) of the samples. Samples obtained from MN chips (each weighing 10–15 mg) were placed in hermetically sealed aluminum pans (TA Instruments, cooled from room temperature to 0 °C at a rate of 10 °C/min and subsequently heated to 200 °C at a heating rate of 10 °C/min). The sample chamber was purged with dry nitrogen at 50 ml/min. Empty pans were used as a reference control. The onset temperature of the discontinuities in the heat flow versus temperature curve was taken as the glass transition point, which was determined using TA Instruments Universal Analysis 2000 software.

### X-ray diffraction

X-ray diffraction (XRD, X’pert Pro Multi-Purpose Diffractometer, PANalytical, Westborough, MA) analysis was used to study structural changes of MN chips stored at specified temperatures over time. The measurements were made in the θ-2θ mode on the MN chip samples using a bracket sample holder with a Cu Kα radiation source (Cu Kα = 1.54059 Å) at room temperature. Data were collected between 2*θ* values of 5° and 50° using a step size of 0.013° and an acquisition time of 30 s per step. Samples were measured at 45 kV and 40 mA. X-ray diffraction patterns were analyzed using Jade 8 software (MDI Materials Data, Livermore, CA) and shown after background correction. Diffraction peaks were fitted to a Gaussian profile.

### Fourier transform infrared spectroscopy

Fourier transform infrared spectrometry (FTIR, Nicolet iS 50 FT-IR, Thermo Fisher Scientific, Waltham, MA) was used to study structural changes of MN chips stored at specified temperatures over time. The FTIR spectra of the samples were recorded at room temperature from 4000 to 600 cm^−1^ and shown after background correction.

### Statistics and Design of Experiments

The design of experiments model for excipient screening was developed using MiniTab software version 18 (MiniTab, State College, PA). Statistics were calculated using either MiniTab or Excel (Microsoft, Redmond, WA). All listed averages represent the arithmetic mean of the samples. Comparison between three or more samples was performed by one-way ANOVA or the data were fit to a general linear model. Comparisons between individual samples were done using an unpaired *t* test. Probability (*p*) values of < 0.05 were considered to be significant.

## Results and discussion

### Excipient screening for IPV stabilization

To identify formulations that stabilize IPV during MN patch fabrication, we studied excipients including maltodextrin, which was previously shown to stabilize IPV [30], and D-sorbitol, sucrose, and trehalose, which are widely used to stabilize proteins during drying or lyophilization [31, 32]. Using a design of experiments model [see Table S1 in Supplemental Information (SI)], we studied the effect of individual excipients as well as combinations of up to four excipients, because multiple stabilizers have often been shown to provide better stability than individual excipients [33].

Trivalent concentrated IPV was formulated with combinations of different excipients in histidine buffer and dried on PDMS chips at 5 °C overnight. The samples were then lyophilized and stored at 25 °C with desiccant for 1 week. Each sample was reconstituted and assayed for D-antigen binding activity by ELISA.

**Fig. 1:**
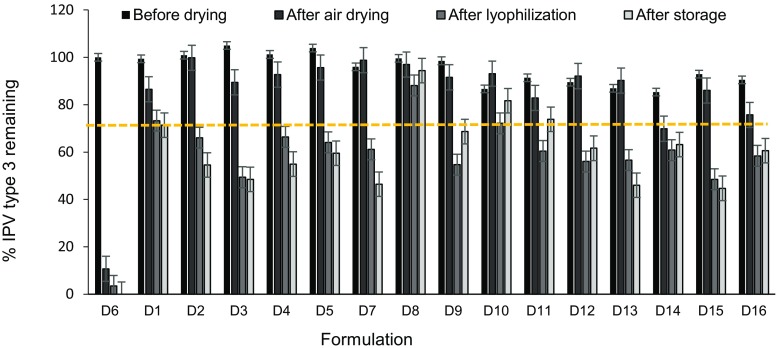
Effect of stabilizing excipients on IPV type 3 activity before drying, after air drying, after subsequent lyophilization and after storage. Vaccine was formulated with combinations of different excipients (each present at a concentration of 5% *w*/*v*) in 0.1 M histidine buffer at pH 6.5 (see Table S1 in SI for key to excipient formulations). In each set of bars, the “before drying” bar shows activity of liquid IPV formulated with excipients before drying. The “after air drying” bar shows IPV activity after air drying at 5 °C with desiccant overnight after casting onto PDMS chips. The “after lyophilization” bar shows IPV activity after lyophilization. The “after storage” bar shows IPV activity after storage of lyophilized vaccine at 25 °C with desiccant for 1 week. The unformulated vaccine control data is shown as D6 bars on the far left. All IPV activity (determined by ELISA) is shown as percentage of the starting stock solutions of concentrated vaccine and a reference line indicating 70% IPV type 3 activity. Asterisk indicates excipient formulation that maintained > 80% activity after drying and storage. Data represent mean ± SEM (standard error of the mean) of *n* = 3 replicates. Companion data on IPV types 1 and 2 are shown in Fig. S1 in SI

In general, varying formulation of IPV type 3 (Fig. 1) and types 1 and 2 (Fig. S1) had little effect of vaccine stability (two-way ANOVA, *p* > 0.05), as determined by D-antigen content measured by ELISA, probably because we selected excipients already shown or expected to stabilize IPV. After air drying, IPV typically lost very little activity (two-way ANOVA, *p* > 0.05), but then lost significant activity after subsequent lyophilization (two-way ANOVA, *p* < 0.004). Sometimes there were additional losses in IPV activity after storage at 25 °C, 0% RH for 1 week, but in most cases post-lyophilization and post-storage activity values were similar (two-way ANOVA, *p* > 0.05). In general, IPV type 2 was most stable, followed by IPV type 1 and IPV type 3 (three-way ANOVA, *p* > 0.05). This is consistent with literature, which reports that IPV type 2 exhibits the best thermostability [34].

It is notable that all three serotypes of unformulated IPV lost ~ 75–90% activity after air drying and lost ~ 90–100% activity after lyophilization (and storage). In contrast, none of the formulated vaccine samples lost more than 60% activity after drying, lyophilization and storage of IPV type 1, 2, or 3. The optimal formulation was the combination of maltodextrin and D-sorbitol (50:50 mass ratio), which maintained at least 80% activity after drying, lyophilization, and storage.

### Buffer and pH screening

**Fig. 2:**
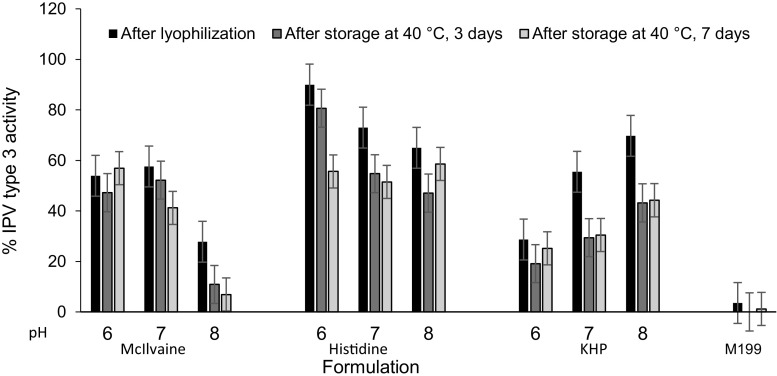
Effect of formulation buffer and pH on IPV type 3 activity after drying on PDMS chips followed by lyophilization and storage. IPV was formulated with 5% *w*/*v* maltodextrin and 5% *w*/*v* D-sorbitol in 0.1 M buffer with pH adjusted using 1 N HCl or 1 N NaOH. Storage was carried out at 40 °C for 3 or 7 days with desiccant. KHP: Potassium hydrogen phthalate, M199: Medium 199. In each set of bars, IPV activity is shown in the first bar after air drying at 5 °C overnight and lyophilization, in the second bar after storage in a desiccator at 40 °C for 3 days and in the last bar after storage at 40 °C in a desiccator for 1 week. Stability of unformulated IPV (i.e., without maltodextrin or D-sorbitol) in M199 media is shown in the bars on the far right. All IPV activity is shown as percentage of IPV activity vaccine casting solution. Data represent mean ± SEM of *n* = 3 replicates. Companion data on IPV types 1 and 2 are shown in Fig. S2 in SI

To assess the effect of buffer and pH, IPV activity was determined after air drying, lyophilization, and storage at 40 °C for 3 days and 7 days using three different buffers and three pH values. Overall, buffer type had a greater effect on activity loss (general linear model, *p* < 0.05) than pH (general linear model, *p* < 0.12), over the range of conditions studied (Figs. 2 and S2 in SI). Histidine buffer maintained activity of type 3 IPV (general linear model, *p* > 0.80) while potassium hydrogen pthalate (KHP) buffer had the most adverse effect (general linear model, *p* < 0.03) (Fig. 2); similar findings were observed in IPV types 1 and 2 (Fig. S2 in SI), although the differences in effects of pH and buffer were less dramatic compared to IPV type 3. Histidine buffer at pH 6 was selected as the best buffer to stabilize all three serotypes. Subsequent MN patch optimization experiments led to 0.35 mM histidine buffer for the first cast formulation and 0.15 mM histidine buffer for the second cast formulation. These buffer concentrations in combination with the other excipients (that can also influence pH) were selected to yield a final pH of 6.3.

### Optimization of polio MN patches

Initial formulation studies above were performed by casting onto flat PDMS chips to facilitate higher throughput. We next assessed formulation optimization in the context of MN patches fabrication by a two-cast process.

#### Optimization of 1st cast formulation

**Fig. 3:**
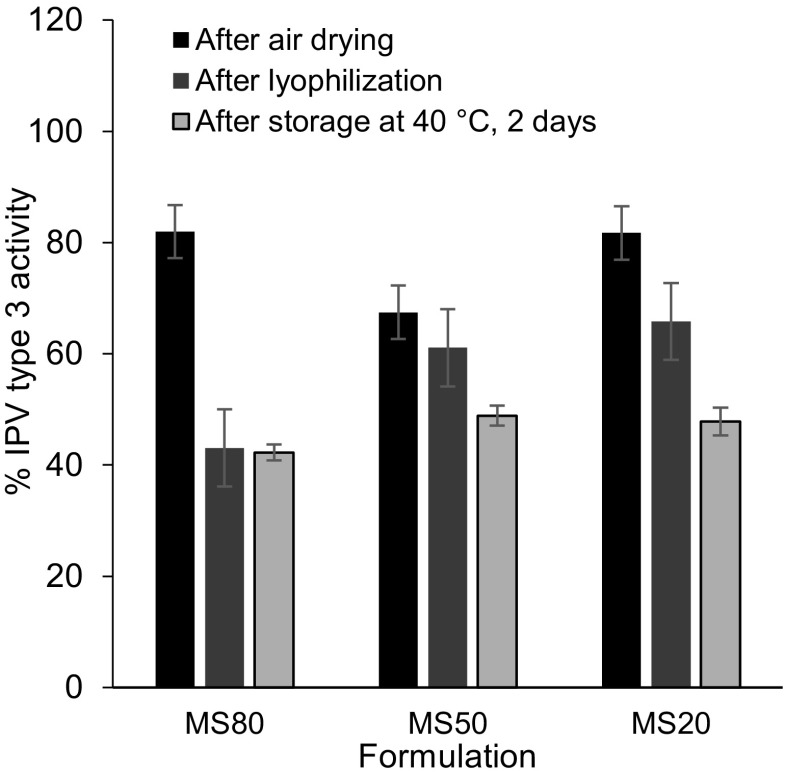
Effect of 1st cast excipient ratio (maltodextrin:D-sorbitol) on IPV type 3 activity after fabricating MN patches, air drying, lyophilization, and storage with desiccant at 40 °C for 2 days. Trivalent IPV was formulated with stabilizing excipients at a total concentration of 10% *w*/*v* in the vaccine casting solution. The maltodextrin:D-sorbitol mass ratios were 80:20 (MS80), 50:50 (MS50), and 20:80 (MS20). The 2nd cast polymer matrix solution consisted of excipients at a total concentration of 45% *w*/*v* fish gelatin and D-sorbitol (at a mass ratio of 78:22) in 0.15 mM histidine buffer at pH 6.3. All IPV activity is expressed as a percentage of IPV activity in the vaccine casting solution. In each set of bars, the first bar shows IPV activity in MN patch after air drying at 5 °C in a desiccator for 2 days; the second bar shows IPV activity after additional lyophilization; and the last bar shows IPV activity after storage at 40 °C for 1 week with desiccant. There was no significant difference between the different ratios of maltodextrin-to-D-sorbitol (two-way ANOVA, *p* > 0.8). Data represent mean ± SEM of *n* = 3 replicates. Companion data on IPV types 1 and 2 are show in Fig. S3 in SI

The first solution cast onto the MN mold contained IPV formulated with maltodextrin and D-sorbitol in different ratios (IPV type 3 in Fig. 3; IPV types 1 and type 2 in Fig. S3 in SI). There was no significant effect of maltodextrin: D-sorbitol ratio on activity of IPV type 1, 2, or 3 (two-way ANOVA, *p* > 0.8). A maltodextrin:D-sorbitol ratio of 20:80 (MS20, on a dry basis, when cast as 10% *w*/*v* solids in solution) was selected as the best formulation because this ratio consistently enabled fabrication of MN patches with sharp microneedle tips.

#### Optimization of 2nd cast formulation

**Fig. 4:**
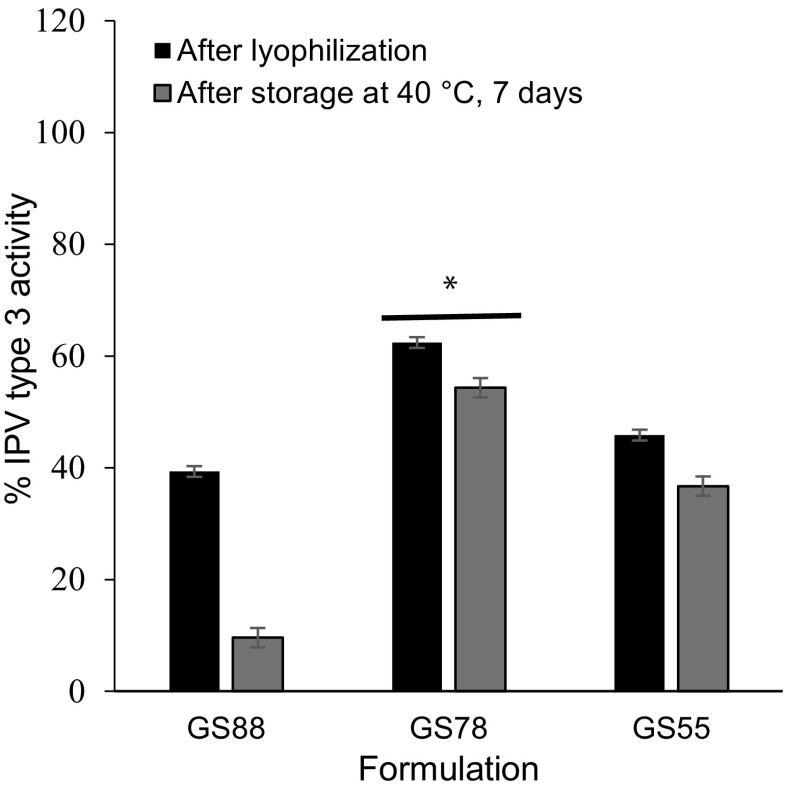
Effect of 2nd cast excipient ratio (fish gelatin-to-D-sorbitol) on IPV type 3 activity after fabricating MN patches, air drying, lyophilization, and storing with desiccant at 40 °C for 7 days. Trivalent IPV was formulated with stabilizing excipients at a total concentration of 10% *w*/*v* in the vaccine casting solution. The maltodextrin:D-sorbitol mass ratios with trivalent IPV was formulated with stabilizing excipients at a total concentration of 10% *w*/*v* in the vaccine casting solution. The maltodextrin:D-sorbitol ratio was 20:80 in 0.35 mM histidine buffer. The 2nd casting solution contained 45% *w*/*v* with varying mass ratios of fish gelatin-to-D-sorbitol in 0.15 mM histidine buffer. The mass ratios used were GS88 (fish gelatin:D-sorbitol = 88:11), GS78 (fish gelatin:D-sorbitol = 78:22), and GS55 (fish gelatin:D-sorbitol = 55:45). All IPV activity is expressed as a percentage of IPV activity in the vaccine casting solution. In each set of bars, the first bar shows IPV activity in MN patch after air drying followed by lyophilization and the second bar shows the IPV activity after storage at 40 °C for 1 week with desiccant. Asterisk depicts a significant difference between IPV activity compared to other two ratios (ANOVA, *p* < 0.05). Data represent mean ± SEM of *n* = 3 replicates. Companion data on IPV types 1 and 2 are show in Fig. S4 in SI

MN patch fabrication involves the addition of a second cast solution which not only forms the MN patch backing but also provides additional mechanical strength to the MNs. To determine the effect of ratio of 2nd cast excipients, MN patches were prepared with a 1st cast consisting of trivalent IPV formulated with maltodextrin and D-sorbitol (20:80) and a second cast solution consisting of varying ratios of fish gelatin to D-sorbitol in 0.15 mM histidine buffer (IPV type 3 in Fig. 4; IPV types 1 and type 2 in Fig. S4 in SI).

No significance differences in IPV type 1 and type 2 activities were observed between GS78 and GS55 formulations (ANOVA, *p* > 0.05), but GS78 was better in stabilizing IPV type 3 (ANOVA, *p* < 0.05). However, MN patches prepared with GS55 formulation were not mechanically strong enough to penetrate the skin. GS88 had adverse effects on all three IPV serotypes (ANOVA, *p* < 0.05), especially after extended storage at 40 °C.

Hence, a fish gelatin:D-sorbitol ratio of 77:22 (on a dry basis, when cast as 45% *w*/*v* solids in solution) was selected as the final 2nd cast formulation because it resulted in the best stabilization of all three IPVs types and produced MN patches with strong microneedle tips.

### Effect of drying conditions

Solid MN patches are formed by drying the cast formulations in the MN mold. As the vaccine is removed from an aqueous environment during drying and subsequent storage, it undergoes changes that can decrease its specific antigenicity [35]. Formulation with polyols such as D-sorbitol and polysaccharides such as maltodextrin, as well as drying at controlled temperature and/or by lyophilization can help maintain antigen activity during drying [36, 37].To study the effect of drying temperature and lyophilization, the residual moisture content of MN patches were measured by TGA and the IPV activity was measured by ELISA after air drying, after subsequent lyophilization, after storage at 40 °C (without lyophilization), and after storage at 40 °C (with lyophilization).

**Fig. 5:**
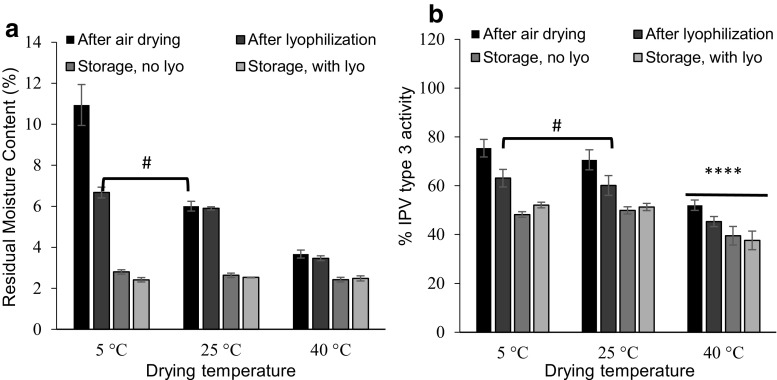
Effect of air drying, subsequent lyophilization and storage conditions on residual moisture content (RMC) of MN patches IPV type 3 activity. **a** Effects on RMC. MN patches were fabricated and air dried at 5 °C, 25 °C, or 40 °C with desiccant for 2 days. Half of the patches were stored at 40 °C for 1 week with desiccant. The other half of the patches were subjected to lyophilization followed by storage at 40 °C for 1 week with desiccant. **b** Effects on IPV type 3 activity. IPV activity is expressed as a percentage of IPV activity in the liquid casting solution. In each set of bars, RMC or IPV type 3 activity is shown in the first bars for MN patches after air drying, in the second bars for MN patches after lyophilization, in the third bars for MN patches stored at 40 °C for 1 week with desiccant (no lyophilization), and in the last bars for MN patches stored at 40 °C for 1 week with desiccant after lyophilization. Hash (#) depicts no statistical difference between the two samples (one-way ANOVA, *p* > 0.05). Asterisk indicates a significant difference in IPV activity (one-way ANOVA, *p* < 0.05). Data represent mean ± SEM of *n* = 3 replicates. Companion data on IPV types 1 and 2 are show in Fig. S5 in SI

The kinetics of water removal from MN patches depended on drying temperature and in some cases, lyophilization, but in all cases the final residual water content after patch storage at 40 °C for 1 week with desiccant was ~ 2.5%, suggesting an equilibrium state of water partitioning between the MN patch, surrounding air and desiccant at that temperature (Fig. 5).

Concerning kinetics, residual moisture content of MN patches depended on temperature during initial air drying with desiccant for 2 days, where drying at 40 °C, 25 °C, and 5 °C resulted in significantly different residual moisture contents of 3.6%, 6.0%, and 10.9%, respectively (one-way ANOVA, *p* < 0.0004) (Fig. 5a). Lyophilization after air drying reduced residual moisture content after air drying at 5 °C (Student’s *t* test, *p* < 0.015), but had no significant effect on residual moisture content after air drying at 25 °C or 40 °C (Student’s *t* test, *p* > 0.7).

Air drying at 5 °C or 25 °C resulted in similar IPV activities (one-way ANOVA, *p* > 0.05), whereas drying at 40 °C resulted in lower IPV activity, especially for IPV types 1 and 3 (one-way ANOVA, *p* < 0.05) (Fig. 5b and Fig. S5 in SI). This is consistent with literature, which reports that heating IPV results in loss of D-antigenicity [38]. After storage at 40 °C for 1 week with desiccant (with or without prior lyophilization), IPV stability was similar when previously air dried at 5 °C or 25 °C, but was lower when previously dried at 40 °C. We therefore selected air drying at 25 °C (since it dries faster than at 5 °C) without lyophilization (since it adds time, cost, and complexity with no apparent advantage in IPV stability) as the preferred MN patch drying method.

### Polio MN patch characterization

**Fig. 6:**
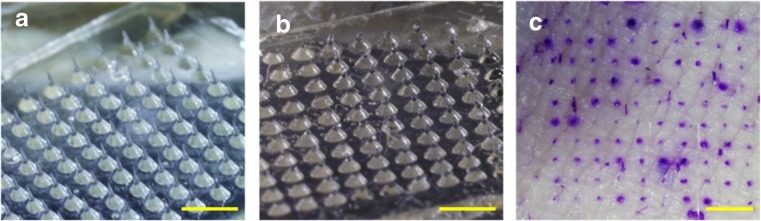
MN patch containing a 10 × 10 array of microneedles was manually inserted into shaved porcine cadaver skin and imaged **a** before insertion and **b** 15 min after insertion to demonstrate the extent of dissolution in the skin. **c** Pig skin stained with gentian violet to reveal sites of skin puncture after MN patch insertion. The scale bars are 5 mm

Based on the optimization of 1st cast solution, 2nd cast solution, and the drying conditions, we fabricated MN patches comprised of a 10 × 10 array of MNs measuring approximately 650 μm in height (Fig. 6a). Upon pressing the MN patches to porcine cadaver skin, leaving them in place for 15 min and then removing them, microscopic imaging of the patch showed that the MNs had substantially dissolved, indicating successful skin penetration and dissolution (Fig. 6b). Each MN (darker color in Fig. 6a, due to optical effect) was mounted on a wide base substrate (lighter color in Fig. 6a) to facilitate MN insertion into skin. While the base substrate remains, the MN is mostly gone. Staining sites of skin penetration further demonstrated that the MNs had punctured the skin (Fig. 6c). These findings show that a formulation optimized for IPV stability was also strong enough to penetrate skin and rapidly dissolve within the skin, thereby generating no sharps waste.

### Long- term stability of polio MN patches

The next step was to determine stability of MN patches during long-term storage up to 1 year at up to 40 °C. Extended storage at elevated temperatures was studied because reduced need for a cold chain of storage is desirable, especially in polio-endemic countries which often have poor infrastructure. Consistent with literature [39],storage of commercial IPV at an elevated temperature of 40 °C led to complete loss of activity within days to weeks (Fig. S6 in SI).

**Fig. 7:**
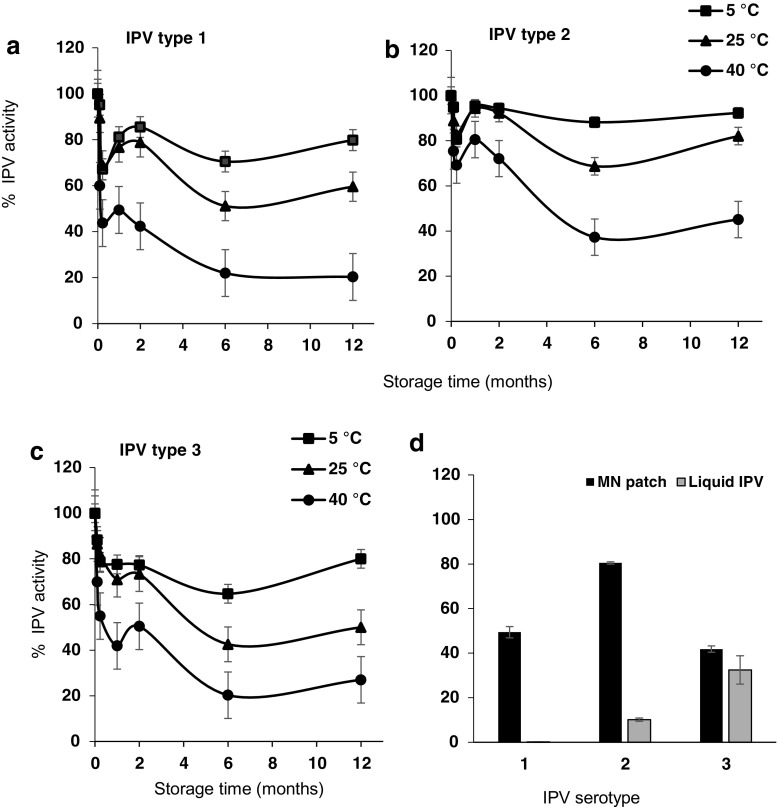
IPV activity in MN patches stored at 5, 25, or 40 °C with desiccant for up to 1 year. MN patches were analyzed using ELISA for **a** IPV type 1, **b** IPV type 2, and **c** IPV type 3 activity at various time points. **d** IPV activity of MN patch and commercially available vaccine, IPOL (Sanofi Pasteur, Rockville, MD), stored for 1 month at 40 °C. IPV activity is expressed as a percentage of IPV activity of MN patches immediately after fabrication or in IPOL as received. Data represent mean ± SEM of *n* = 3 replicates

MN patches, however, were able to retain IPV activity much better than liquid vaccine, especially at elevated storage temperatures. All three IPV serotypes maintained > 70% activity after 2 months and > 50% activity after 1-year storage at 5 °C or 25 °C with desiccant (Fig. 7). It is notable that stability at 25 °C was as good as 5 °C (two-way ANOVA, *p* > 0.2). Storage at 40 °C was not as good as at 5 °C (two-way ANOVA, *p* = 0.004) or 25 °C (two-way ANOVA, *p* = 0.002). Storage at 40 °C yielded > 40% activity after 2 months and > 20% activity after 1 year for all three serotypes. We are not sure why IPV stability at the 1-week time point was lower than the overall trend would suggest, and consider it might be due to measurement variability, although additional studies are needed to investigate these effects in greater detail.

### Correlation of residual moisture content in patch to IPV stability

**Fig. 8:**
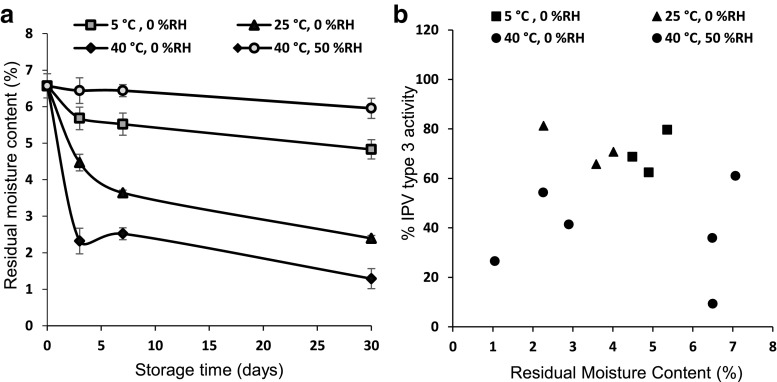
**a** Effect of storage conditions on residual moisture content of polio MN patches stored at 5, 25, or 40 °C with desiccant or at 40 °C and 50% RH. **b** Correlation of IPV type 3 stability in MN patches to residual moisture content of the patches stored at 5, 25, or 40 °C with desiccant or at 40 °C and 50% RH. Regression analysis showed an R^2^ value of 0.009, indicating poor correlation. Data represent mean ± SEM of *n* = 3 replicates. Companion data on IPV types 1 and 2 are show in Fig. S7 in SI

Our studies so far indicated that IPV instability was driven by elevated temperature (especially at 40 °C). However, IPV instability could also be caused by presence of water, which is a potent plasticizer because of its low glass transition temperature (T_g_ = − 135 °C) [40]. Hence, residual moisture in the MN patch can facilitate reactions controlled by molecular mobility, such as protein unfolding, aggregation, and chemical degradation. We therefore prepared and stored MN patches at various temperatures and humidity levels to generate patches with a range of residual moisture content (Fig. 8 and Fig. S7 in SI).

Residual moisture content of MN patches immediately after fabrication was 6.6 ± 0.2%. Storage at different temperatures for different times produced patches with residual moisture contents as low as 1.3 ± 0.1%. Storage at 5, 25, or 40 °C for 1 month with desiccant resulted in moisture loss of 2%, 4%, or 5%, respectively. Storage at 40 °C and 50% RH resulted in minimal moisture loss of 0.5%.

At these different times and temperatures, IPV activity loss ranged between 91 and 7%. Correlation between IPV activity and residual moisture content (independent of temperature) was poor (Fig. 8b and Fig. S7 in SI), with *R*^2^ values of < 0.05. In contrast, IPV activity and temperature (independent of residual water content) were correlated (*R*^2^ values of 0.38–0.67) (Fig. S8 in SI). This shows that IPV destabilization processes are driven by elevated temperature with little influence from residual moisture content over the range of conditions studied.

### Characterization of changes in MN matrix material properties during patch storage

We determined the composition of MNs (i.e., without the patch backing) to determine the effect of IPV-excipient interaction in IPV activity loss. We then studied the changes in material properties in MNs during storage at 5 °C, 25 °C, or 40 °C with desiccant and then characterized them as described below.

#### Determination of glass transition temperature of MNs by DSC

**Fig. 9:**
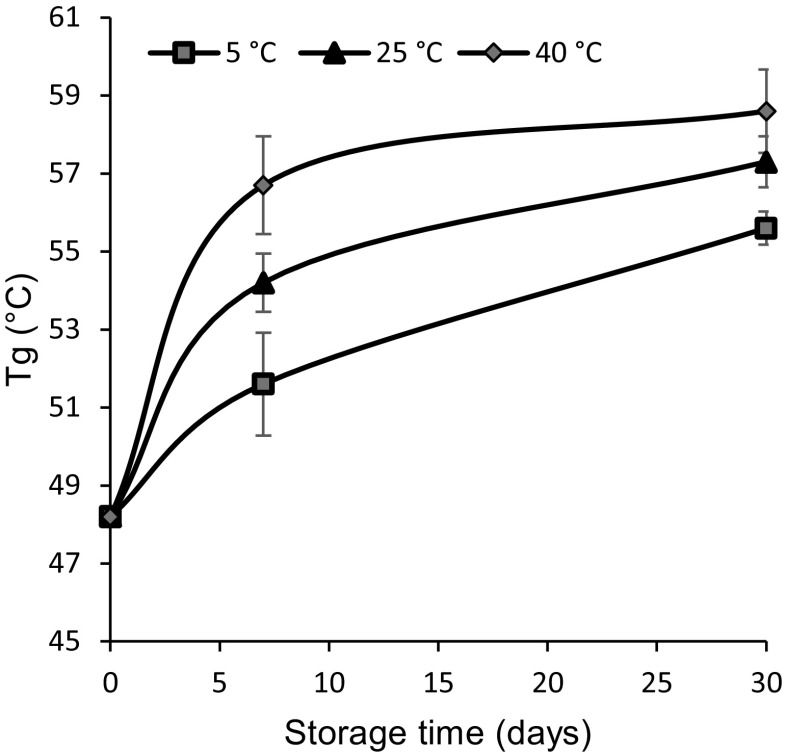
Effect of storage time and temperature on T_g_ of MN matrix material measured by DSC. T_g_ was measured at specific times for up to 30 days at 5 °C, 25 °C, or 40 °C with desiccant. Data represent mean ± SEM of *n* = 3 replicates

MNs stored below their T_g_ will be in an amorphous glass phase, which can cause IPV instability by retarding vaccine mobility, interactions, and prevent crystallization [41]. Using DSC, we found that the MN matrix initially had a T_g_ of 48 °C, which increased over time to as much as 59 °C. This finding is consistent with our prior observation that residual moisture content decreased with storage time (Fig. 8a), since a decrease in moisture content is generally associated with an increase in T_g_ [42]. These data may also explain why stability at 5 °C and 25 °C are similar, since they are both well below the T_g_, but stability was worse at 40 °C, which is closer to the T_g_ of the MNs (Fig. 9).

#### Determination of chemical composition changes of MNs by FTIR

**Fig. 10:**
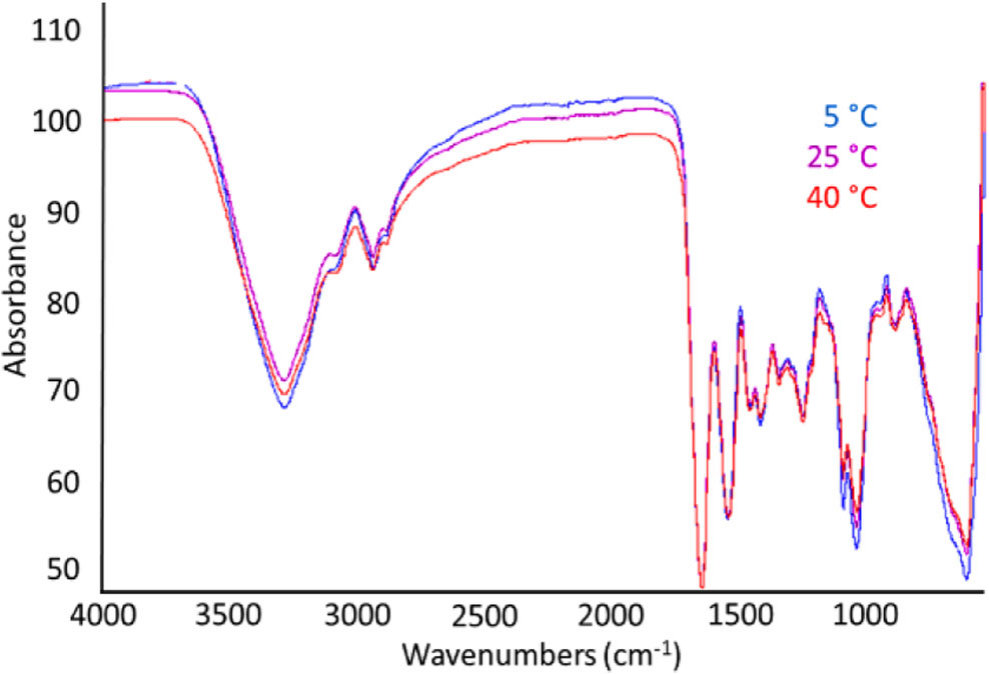
FTIR spectrum of MN matrix samples measured 30 days after storage at 5 °C, 25 °C, or 40 °C with desiccant. Data represents averages of *n* = 3 replicates

We next used FTIR to study possible changes in molecular structure, and chemical bonding in the MN matrix after 30 days of storage at 5 °C, 25 °C, or 40 °C (Fig. 10). We interpret the peaks situated at 3272–3370, 1627–1632, 1532–1546, and 1235–1276 cm^−1^ to correspond to N-H stretching and free water, amide I, amide II, and amide III, respectively [43]. We further interpret amide I to represent C=O stretching/hydrogen bonding coupled with COO; amide II to represent bending vibration of N–H groups and stretching vibrations of C-N groups; and amide-III to be related to the in-plane vibrations of C-N and N-H groups of bound amide or vibrations of CH_2_ groups of proline side chains in gelatin [44]. The strong peak between 4000 and 3200 cm^−1^ and the peak at 1081 cm^−1^ are believed to be mainly from –OH stretching and C-O stretching vibrations from D-sorbitol and maltodextrin, respectively [45, 46]. The spectra appear very similar, which means that chemical changes were not detected among the samples. In contrast, the long-term stability study showed greater IPV instability at 40 °C when compared to 5 °C and 25 °C. Hence, the lack of changes in molecular level interactions between IPV and excipients determined by FTIR could not be correlated to IPV instability.

#### Determination of matrix structural changes in MN by XRD

**Fig. 11:**
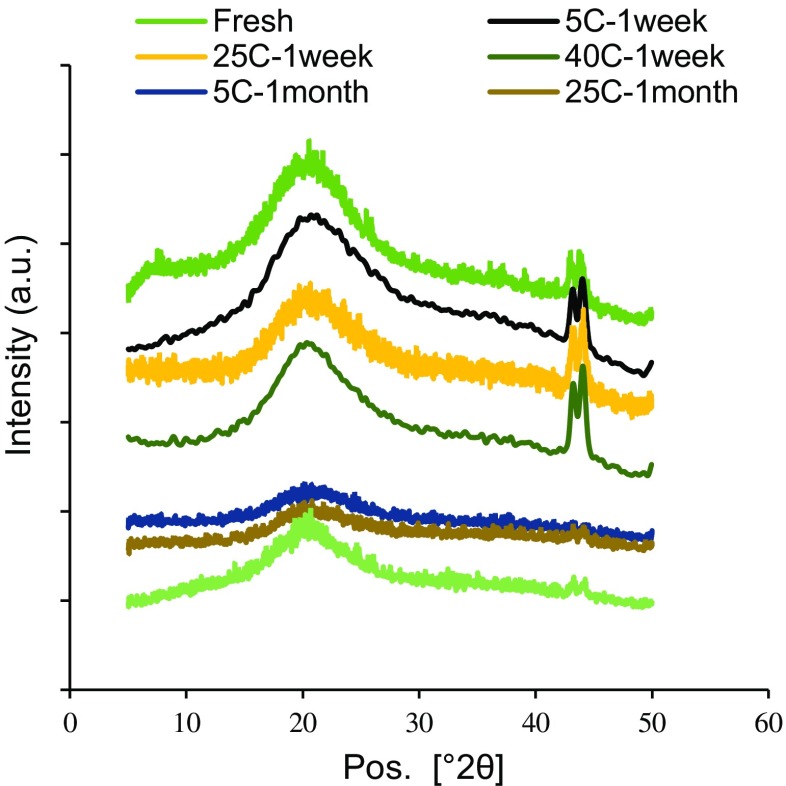
X-ray diffraction patterns corresponding to MN matrix samples stored at 5 °C, 25 °C, or 40 °C for up to 30 days. Data represent averages of *n* = 3 replicates

XRD was performed to better understand structural changes in the MN matrix (Fig. 11). The broad peak centered near 2*θ* = 20° observed in all samples indicates that the MN matrix is in an amorphous phase [47], which we interpret to be mainly composed of gelatin and maltodextrin based on the XRD patterns of the individual materials (Fig. S8 in SI). The fresh and 1-week samples also show a sharp peak at approximately 2*θ* = 42–43°, which suggest an additional crystalline phase probably composed of D-sorbitol. The intensities and peak areas for fresh and 1-week samples were similar, independent of the storage temperature. After 1 month, however, the crystalline peak heights for all the samples decreased by ~ 76% and the peak areas decreased by ~ 83%. This suggests that the MN matrix became more amorphous during storage, as residual water content decreases and T_g_ increases independent of the storage temperature. Since the long-term stability study showed greater IPV instability at elevated temperature, we conclude that the changes observed in the bulk matrix properties determined by XRD could not be correlated to IPV instability.

## Discussion

The WHO has recommended replacing OPV with IPV worldwide to eliminate the risk of vaccine-derived polioviruses [9]. However, some limitations with the current IPV vaccination are the need for trained healthcare personnel, risk of needle stick injuries [48], and the need for cold chain to maintain IPV potency. MN patches can improve IPV delivery by enabling simple administration by minimally trained personnel and avoiding generation of biohazardous sharps waste [49]. In this study, we optimized MN patch formulation to improve IPV stability during extended storage at elevated temperatures.

Initial formulation optimization studies showed that a combination of maltodextrin and sorbitol was optimal for minimizing IPV loss during drying. Buffer screening showed that histidine buffer at pH 6 was optimal for IPV stability. Guided by these findings, MN patch optimization studies identified a 1st cast composition of maltodextrin:D-sorbitol ratio of 20:80 (on a dry basis) and a 2nd cast composition of fish gelatin:D-sorbitol ratio of 77:22 (on a dry basis, when cast as 45% *w*/*v* solids in solution) that provided the best stabilization of all three types of IPVs and produced MN patches with strong microneedle tips. Drying temperature optimization studies showed that air drying of the MN patches at 25 °C was effective, and subsequent lyophilization did not show any significant improvement in IPV stability.

The main components of the MN patch fabrication are: preparation of two casting solutions, sequential casting of the solutions, drying and packaging. With further development, this process could be scaled up to meet the potential demands for many millions of patches per year required by the polio eradication program. The IPV patches were tested in pig cadaver skin and found to insert into skin by thumb without the need for an applicator. This simple application process could enable administration by minimally trained personnel [50, 51] thereby reducing costs by reducing the need for healthcare professionals. This simplified administration could facilitate more efficient mass IPV vaccination campaigns and routine IPV vaccination too. The inserted patches also showed near-complete MN dissolution in the skin within 15 min. This MN dissolution has the potential to eliminate the risks of biohazardous sharps. Finally, the MN patch demonstrated significant thermostability even at 25 °C for up to 1 year (> 50% activity) or at 40 °C for up to 2 months (> 40% activity). This thermostability could aid reduce reliance on cold chain storage, especially for short periods of time (days to weeks) during vaccination campaigns and transport to remote locations. This collection of capabilities represents a significant advancement that can overcome many of the hurdles posed by the transition from OPV to IPV. Future studies will need to assess mechanical properties of MN patches, IPV immunogenicity in animals and people, and other quality attributes after extended storage. Although not addressed in this study, vaccination by MN patch has enables dose sparing for some vaccines [52, 53], including IPV [54], which could enable vaccine costs savings. Remaining questions include determining the cost of IPV vaccination by MN patch [55], the possibility of skin vaccination by MN patch inducing mucosal immunity [56] and the number of vaccine doses needed for protective immunity.

Mechanistic studies were performed to understand the effect of MN matrix properties on IPV activity during storage. Residual moisture content of the patches decreased with increase in storage temperature but no correlation was seen between moisture content and IPV stability. MN patches showed an increase in T_g_ with increase in storage temperature, which was consistent with the moisture loss determined by TGA (i.e., where moisture loss correlates with increased T_g_). This suggests that the MN matrix become more amorphous at elevated storage temperature and over time. XRD results also showed an increase in amorphousness of the matrix upon extended storage_._ Even though a more amorphous (i.e., glassy) matrix should retard diffusion-controlled reactions such as protein unfolding, and aggregation, IPV was less stable at elevated temperature and longer storage times (both of which promote diffusion-controlled, and most non-diffusion-controlled reactions). Because none of these trends in macroscopic MN matrix changes correlated with changes in IPV activity, we hypothesize that intramolecular interactions in IPV virus particles and inter molecular IPV-excipient interactions could be the source of IPV instability.

## Conclusions

This study presents the development of a dissolving MN patch for IPV vaccination. Formulation and process optimization studies were performed to identify an excipient combination and drying condition during MN patch fabrication that minimized IPV activity loss. The MN patch was simple to administer, generated no sharps waste, and maintained significantly improved thermostability during extended storage at elevated temperature compared to conventional liquid IPV. When stored with desiccant, IPV in MN patches maintained > 70% activity after 2 months and > 50% activity after 1 year at 5 °C or 25 °C, and maintained > 40% activity after 2 months and > 20% activity after 1 year at 40 °C. Changes in MN matrix properties such as residual moisture content, T_g_ and crystallinity were measured, but did not correlate with changes in IPV activity. These findings suggest that a MN patch could enable increased thermostability and other advantages important to IPV vaccination and eradication programs.

## Electronic supplementary material

ESM 1 (PDF 817 kb)
