# Supplementary material for: Development of a thermostable microneedle patch for polio vaccination
Source: Drug Deliv Transl Res. 2018 Dec 12;9(1):192–203. doi: 10.1007/s13346-018-00608-9 (PMC6328527; doi:10.1007/s13346-018-00608-9)
Supplement: Supplementary file 1 — (PDF 817 kb) [file 13346_2018_608_MOESM1_ESM.pdf]

Development of a thermostable dissolving microneedle patch for polio vaccination

Chandana Kolluru, Yasmine Gomaa, Mark R. Prausnitz

Supplemental figures

3.1 Excipient screening for IPV stabilization

Table S1: Design of Experiments table showing excipient combinations tested for IPV stabilization[1]

	Sorbitol (%)	Sucrose (%)	Trehalose (%)	Maltodextrin (%)
D1	5	0	5	0
D2	0	5	5	5
D3	0	5	5	0
D4	5	5	0	5
D5	0	0	5	5
D6	0	0	0	0
D7	0	0	5	0
D8	5	0	0	5
D9	5	5	5	5
D10	5	0	5	5
D11	5	5	5	0
D12	0	5	0	5
D13	0	0	0	5
D14	5	5	0	0
D15	0	5	0	0
D16	5	0	0	0

[1]The left column shows the formulation number and the other columns show the composition of the formulation on a percent w/v basis in 150 mM histidine buffer at pH 6.5.

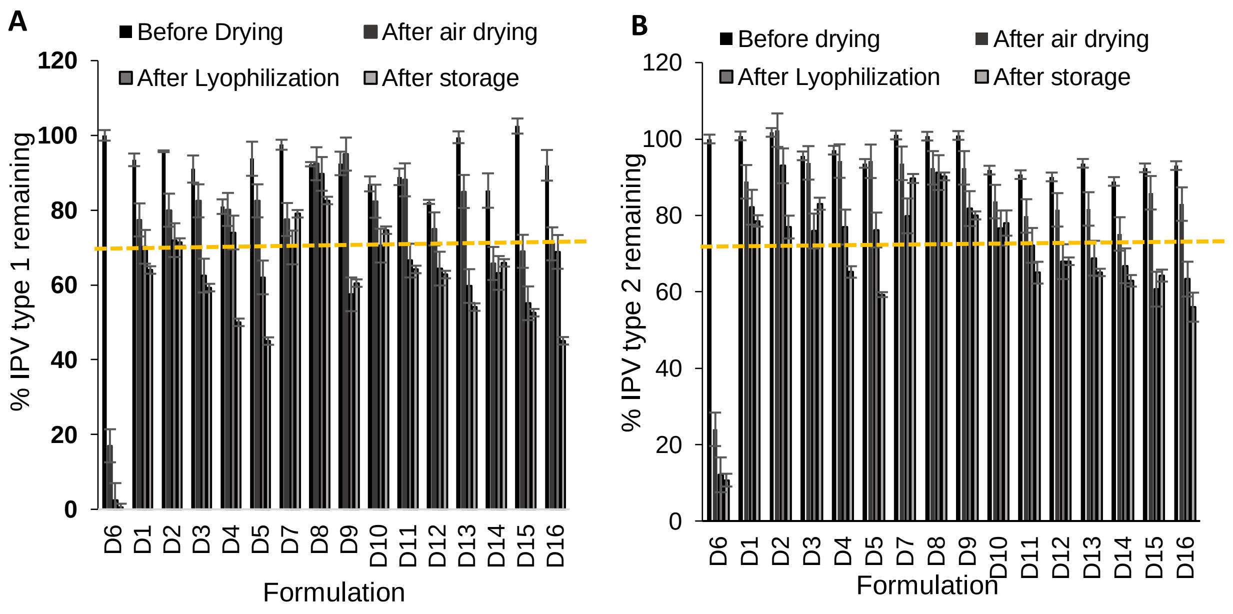

Fig. S1 Effect of stabilizing excipients on (A) IPV type 1 and (B) IPV type 2 activity before drying, after air drying, after subsequent lyophilization and after storage. Vaccine was formulated with combinations of different excipients (each present at a concentration of 5% w/v) in 150 mM histidine buffer at pH 6.5 (see Table S1 for key to excipient formulations). In each set of bars, the first bar shows activity of liquid IPV formulated with excipients before drying. The second bar shows IPV activity after air drying at 5°C with desiccant overnight after casting onto PDMS chips. The third bar shows IPV activity after lyophilization. The fourth bar shows IPV activity after storage at 25 °C with desiccant for 1 week. The unformulated vaccine control data is shown as D6 bars on the far left. All IPV activity (determined by ELISA) is shown as percentage of concentrated stock vaccine solution. Asterisk (*) indicates combination excipient formulation which maintained >80% activity drying and storage. Data represent mean ± SEM (standard error of the mean) of n= 3 replicates

3.2 Buffer and pH Screening

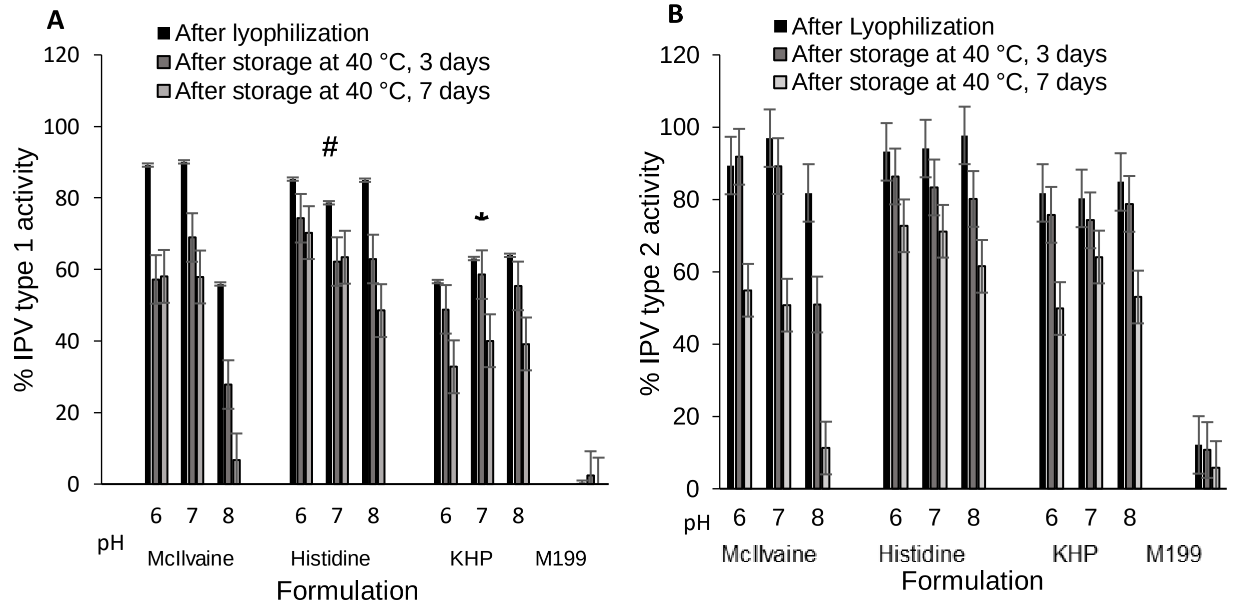

Fig. S2. Effect of formulation buffer and pH on (A) IPV type 1 and (B) IPV type 2 activity after air drying on PDMS chips, after subsequent lyophilization and after storage. IPV was formulated with 5% w/v maltodextrin and 5% w/v D-sorbitol in 0.1 M buffer with pH adjusted using 1 N HCl or 1 N NaOH. Storage was carried out at 40 ˚C for 3 or 7 days with desiccant. KHP: Potassium hydrogen pthalate, M199: Medium 199. In each set of bars, IPV activity (determined by ELISA) is shown in the first bar after air drying at 5°C overnight and lyophilization, in the second bar after storage in a desiccator at 40 ˚C for 3 days and in the last bar after storage at 40 ˚C in a desiccator for 1 week. Stability of unformulated IPV (i.e., without maltodextrin or D-sorbitol) in M199 media is shown in the bars on the far right. Hash (#) represents buffer which did not significantly affect IPV activity compared to activity in the vaccine casting solution (general linear model, $p > 0.80$). Asterisk (*) represents buffer which had the most adverse effect on IPV stability compared to activity in the vaccine casting solution (general linear model, $p < 0.03$). All IPV activity is shown as percentage of activity in the vaccine casting solution. Data represent mean ± SEM (standard error of the mean) of n= 3 replicates

3.3 Optimization of IPV MN patches

3.3.1 Optimization of 1st cast formulation

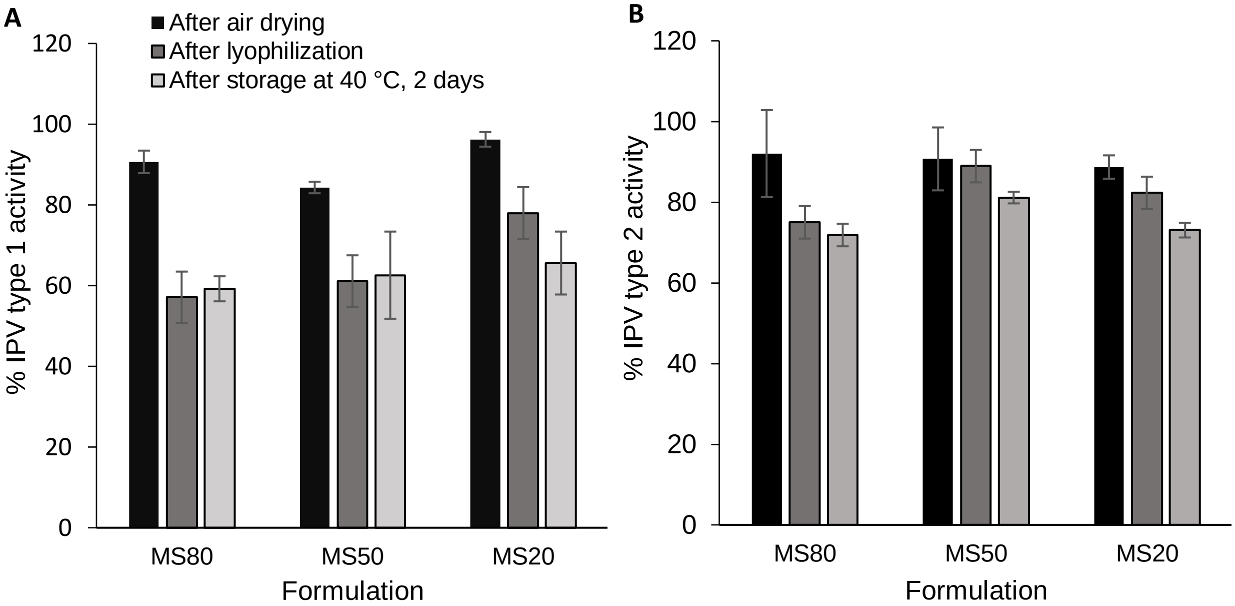

Fig. S3 Effect of 1st cast excipient ratio (maltodextrin to D-sorbitol) on (A) IPV type 1 and (B) IPV type 2 activity after fabricating MN patches, air drying, subsequent lyophilization and storage with desiccant at 40 °C for 2 days. Trivalent IPV was formulated with stabilizing excipients at a total concentration of 10 w/v% in the vaccine casting solution. The mass ratios used were MS80 (maltodextrin: D-sorbitol 80:20), MS50 (maltodextrin: D-sorbitol 50:50) and MS20 (maltodextrin: D-sorbitol 20:80). The 2nd cast polymer matrix solution consisted of 45 wt% fish gelatin to D-sorbitol (78:22) in 0.15 mM histidine buffer. All IPV activity (determined by ELISA) is shown as percentage of vaccine activity in the casting solution. In each set of bars, the first bar shows IPV activity in MN patch after air drying at 5°C in a desiccator for 2 days, the second bar shows IPV activity after additional lyophilization and the last bar shows the IPV activity after storage at 40 °C for 1 week with desiccant. There was no significant difference between the different ratios of maltodextrin to D-sorbitol (Student's t-test, $p > 0.05$). Data represent mean ± SEM (standard error of the mean) of n= 3 replicates

3.3.2 Optimization of second cast

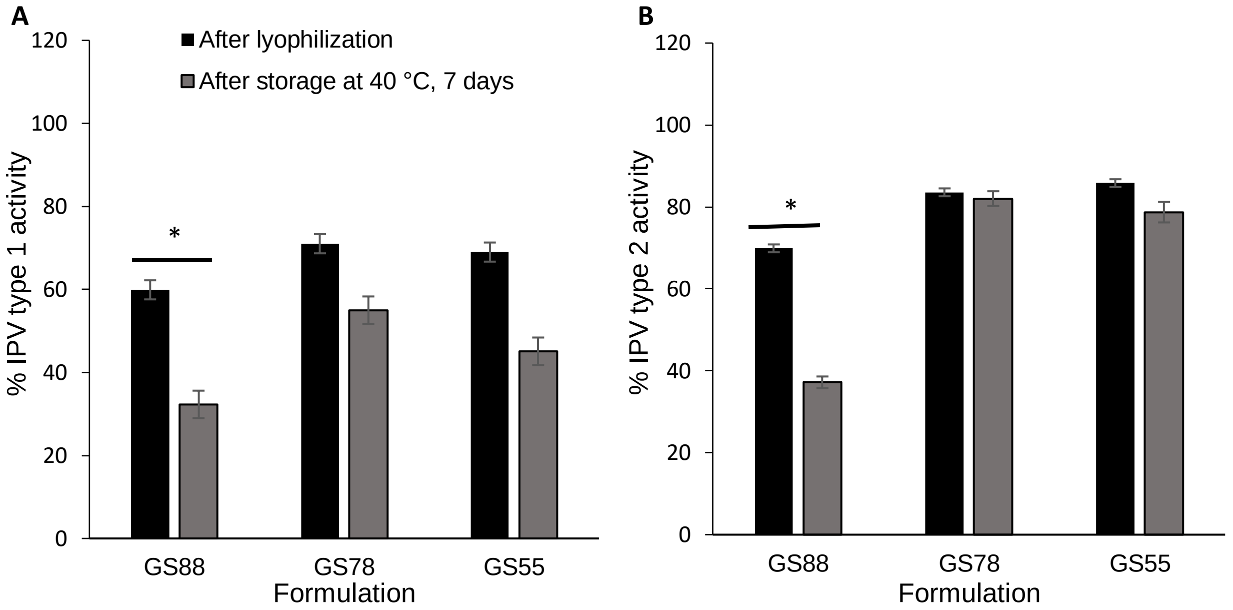

Fig. S4 Effect of 2nd cast excipient ratio (fish gelatin to D-sorbitol) on (A) IPV type 1 and (B) IPV type 2 activity after fabricating MN patches, air drying, further lyophilization and storing with desiccant at 40 °C for 7 days. Trivalent IPV was formulated with 10 w/v% casting solution of maltodextrin: D-sorbitol (20:80) and a 45 wt% 2nd cast solution consisting of varying mass ratios of fish gelatin to D-sorbitol. GS88 (fish gelatin: D-sorbitol 88:11), GS78 (fish gelatin: D-sorbitol 78:22) and GS55 (fish gelatin: D-sorbitol 55:45) in 0.15 mM histidine buffer. All IPV activity (determined by ELISA) is expressed as a percentage of IPV activity in the vaccine casting solution. In each set of bars, the first bar shows IPV activity in MN patch after air drying followed by lyophilization and the second bar shows the IPV activity after storage at 40 °C for 1 week with desiccant. Asterisk (*) indicates a significant difference between the IPV activity compared to other samples (Student's t test, $p < 0.05$). Data represent mean ± SEM (standard error of the mean) of n= 3 replicates

3.3.3 Effect of drying conditions

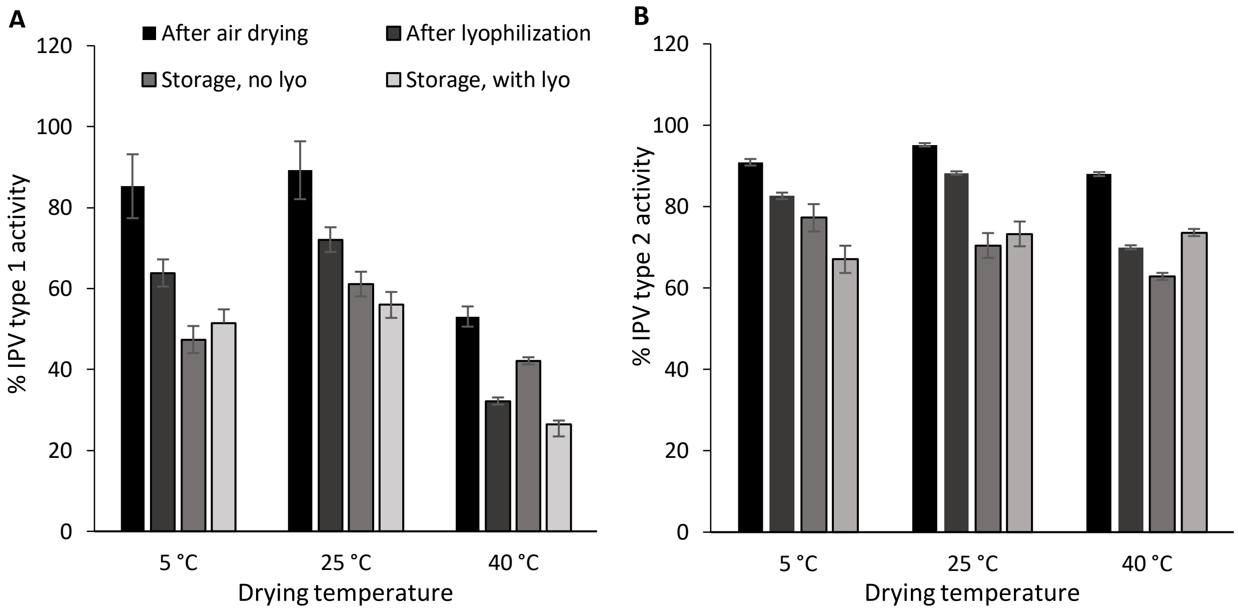

Fig. S5 Effect of air drying, subsequent lyophilization and storage conditions on (A) IPV type 1 and (B) IPV type 2 activity of MN patches air dried at 5 °C, 25 °C or 40°C with desiccant. Trivalent IPV was formulated with 10 w/v% casting solution of maltodextrin: D-sorbitol (20:80) and a 45 wt% 2nd cast solution consisting of fish gelatin to D-sorbitol (78:22). All IPV activity (determined by ELISA) is expressed as a percentage of IPV activity in the vaccine casting solution. In each set of bars, the first bar shows IPV type 2 activity in MN patch after air drying, the second bar shows the activity after subsequent lyophilization, the third bar shows activity for MN patches storage at 40 °C for 1 week with desiccant (no lyophilization) and the last bar shows the activity for MN patches storage at 40 °C for 1 week with desiccant after lyophilization. Data represent mean ± SEM (standard error of the mean) of n= 3 replicates

3.4 Long term stability of polio MN patches

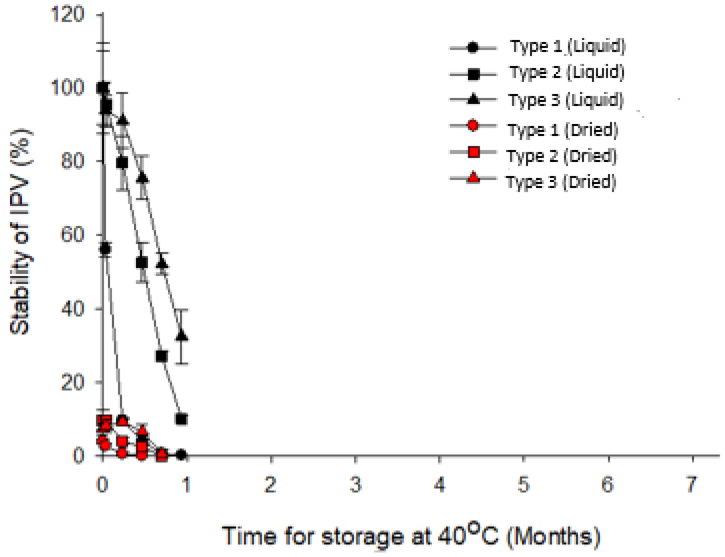

Fig. S6 Effect of storage temperature on IPV types 1, 2 and 3 activities of commercially available vaccine, IPOL® Sanofi Pasteur, Rockville, MD) in liquid and dried form when stored with desiccant for up to 1 months at 40 ˚C. Data represent mean ± SEM (standard error of the mean) of n= 3 replicates.

3.5 Correlation of residual moisture content in patch to IPV stability

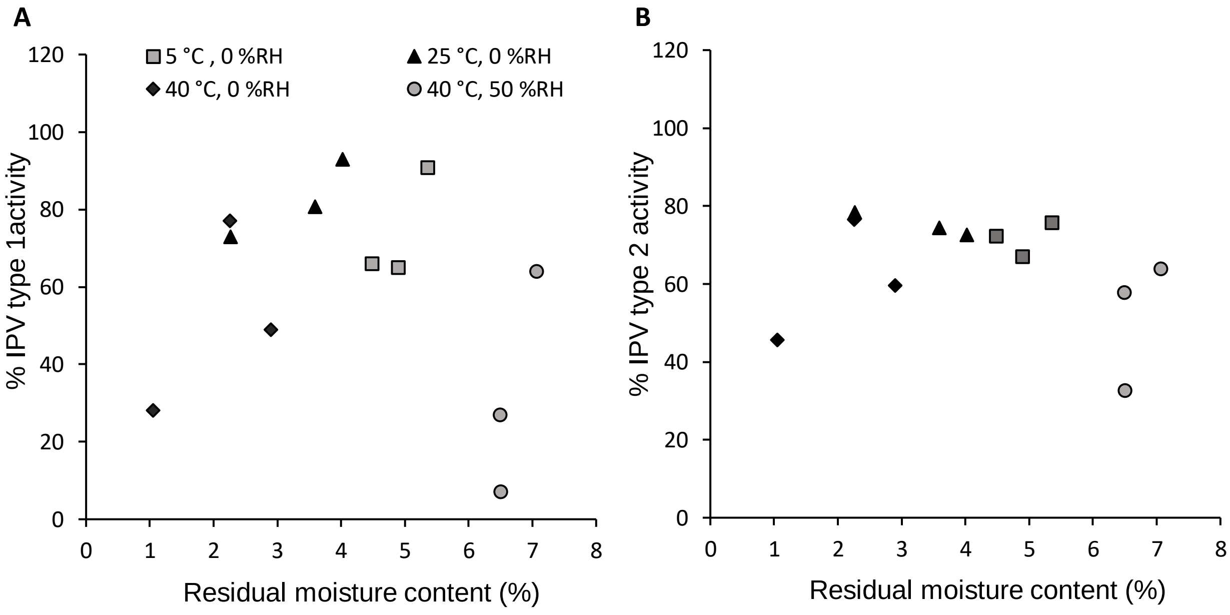

Fig. S7 Correlation of (A) IPV type 1 and (B) IPV type 2 activity in MN patches to RMC of the patches measured after storage at 5, 25 or 40 °C with desiccant or after storage at 40 °C and 50% relative humidity (RH). Regression

analysis showed an R^2 value of 0.032 and 0.049 for IPV type 1 and IPV type 2, respectively. Data represent mean ± SEM (standard error of the mean) of n= 3 replicates

3.6 Correlation of IPV stability to storage temperature

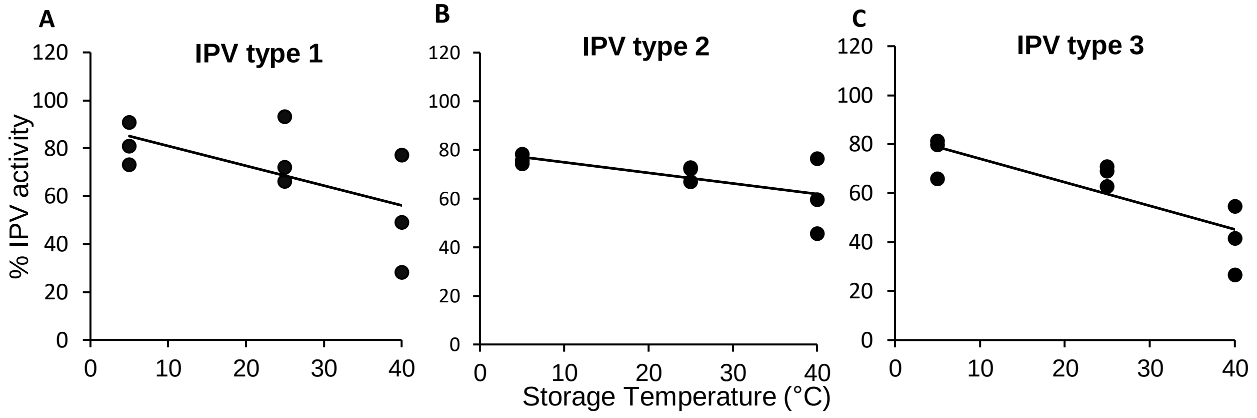

Fig. S8 Correlation of (A) IPV type 1 and (B) IPV type 2 (C) IPV type 3 activity in MN patches to storage temperature of 5, 25 or 40 °C with desiccant. Regression analysis showed an R^2 value of 0.38, 0.39 and 0.67 for IPV type 1, IPV type 2, and IPV type 3 respectively. Data represent mean ± SEM (standard error of the mean) of n= 3 replicates.

3.7 XRD patterns for raw materials used for MN matrix patch preparation

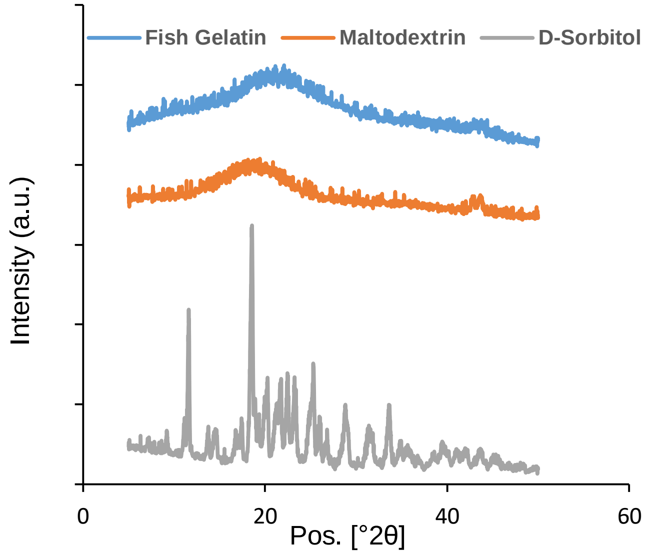

Fig. S9 Representative X-ray diffraction (XRD) patterns corresponding to fish gelatin, maltodextrin and D-sorbitol used in preparation of MN matrix samples
